# Dynamic contagion potential framework for optimizing infection control in healthcare

**DOI:** 10.3389/fpubh.2025.1566854

**Published:** 2025-07-31

**Authors:** Alexandra Fedrigo, Mohamad Nassar, Jennifer Bail, Antonia Bates-Ford, Satyaki Roy

**Affiliations:** ^1^Department of Mathematical Sciences, The University of Alabama in Huntsville, Huntsville, AL, United States; ^2^Department of Electrical & Computer Engineering and Computer Science, University of New Haven, West Haven, CT, United States; ^3^Department of Nursing, The University of Alabama in Huntsville, Huntsville, AL, United States

**Keywords:** hospital-acquired infections, infection control, contagion potential, optimization, resource allocation

## Abstract

**Introduction:**

Hospital-acquired infections (HAIs) caused by bacterial and viral pathogens continue to affect millions annually, placing a persistent burden on healthcare systems. Traditional infection control strategies often fall short due to their inability to assess real-time spatial and movement data within healthcare environments dynamically. This study addresses that gap by leveraging the concept of *contagion potential* (CP), a behavior- and context-driven metric of infection risk, to develop a framework for minimizing the incidence of HAIs.

**Methods:**

The proposed framework integrates CP, which encapsulates an individual's susceptibility and transmissibility, taking into account movement patterns and interactions across hospital units. Unlike models requiring precise tracking, this approach uses coarse location data to construct a dynamic infection risk landscape. CP parameters are continuously learned and updated over time through behavioral data, enabling real-time risk inference. The framework also introduces a CP-based optimization algorithm for patient-to-unit assignments that jointly minimizes contagion risk while satisfying clinical and logistical constraints.

**Results:**

The framework's efficacy is validated through modular and integrated evaluations. Simulations incorporate mobility patterns reflecting homogeneous and heterogeneous mixing, with infection spread following empirically grounded transmission models. Results demonstrate that incorporating CP significantly reduces infection propagation, enhances patient safety, and leads to more efficient healthcare resource allocation.

**Discussion:**

This study presents a dynamic, data-driven framework for infection control within healthcare facilities. By incorporating behavior-aware contagion metrics into patient flow decisions, the approach offers a scalable and proactive infection prevention strategy. The findings underscore the potential of CP to improve both operational outcomes and patient well-being in healthcare environments.

## 1 Introduction

Reports from the Centers for Disease Control and Prevention highlight a substantial public health issue, with millions of hospital-acquired infections (HAIs) annually, primarily bacterial, contributing to nearly 100,000 deaths each year (1, 2). This situation presents a significant burden on healthcare systems, both economically and in terms of patient safety. In response, there has been an increasing emphasis on clinical interventions aimed at reducing the incidence of specific HAIs, including surgical site infections (SSI), ventilator-associated pneumonia (VAP), and central line-associated bloodstream infections (CLABSI) (3–5). As healthcare institutions strive to alleviate the impact of HAIs, there is a growing consensus on the importance of research initiatives that utilize available data to create integrated strategies for prevention and management, with the ultimate goal of improving patient outcomes and healthcare efficiency (6).

The COVID-19 pandemic has further exposed the susceptibility of critically ill patients to HAIs, such as VAP and bloodstream infections (7). These challenges, exacerbated by shortages in staffing and supplies, have imposed additional pressure on healthcare systems (8). The pandemic has also amplified concerns among healthcare workers (HCWs) about transmitting infections to their immediate patients and families, leading many to self-isolate, which has negatively impacted both workforce availability and mental health (9). While measures such as *patient isolation* and *frequent testing* have been employed to safeguard HCWs from infection (10), there remains a critical need for data-driven contagion control measures and longitudinal studies within healthcare environments (11, 12). Studies on HAIs reveal a strong correlation with invasive devices, emphasizing the need for stratification to model infection dynamics within medical-surgical intensive care units (13–16). These insights underscore the urgency of strategies that minimize the risks of HAIs, protecting both patients and HCWs from diseases like multidrug-resistant organisms (MDROs) and *Clostridioides difficile* (*C. diff* ).

Patient allocation and scheduling in healthcare systems have been studied, primarily focusing on two key areas: (1) patient referral to clinics and timeslot assignment and (2) human and clinical resource scheduling based on workload and expertise. In the first line of research, patient referral models explore strategies for allocating patients among healthcare facilities to optimize resource utilization and minimize waiting times. Simulation-optimization methods have been developed to address uncertainties in patient arrival times and medical operation durations, integrating heuristic algorithms with particle swarm optimization to enhance referral efficiency (17). Studies have also emphasized the significance of communication and coordination between primary care physicians and specialists, showing that improved collaboration enhances referral completion rates and physician satisfaction (18). Additionally, clustering-based approaches such as Fuzzy C-Means have been proposed to optimize patient referrals and scheduling, cost, and waiting times (19).

Beyond referrals, the problem of nurse-to-patient assignment in homecare has been explored, where analytical structural policies help balance workloads and continuity of care (20). Parallel to patient allocation, research on scheduling healthcare personnel has gained attention in optimizing nurse schedules to balance workload. Integer programming and evolutionary algorithms have been employed to solve nurse scheduling problems, showing improvements in algorithmic performance and practical feasibility (21). Genetic algorithms have also been applied to nurse scheduling, utilizing indirect coding and heuristic decoders to construct efficient schedules while overcoming algorithmic constraints (22). Collectively, these studies contribute to an understanding of patient and resource allocation, offering methodologies to enhance health services. *There exist limited efforts that jointly address the challenge of inpatient assignment in a manner that simultaneously curbs HAIs while meeting both logistical and clinical demands*.

For this study, the term *contagion* refers to the transmission of infectious agents through close contact between individuals. Previous works have employed contact networks derived from precise mobility data, such as Global Positioning System or Bluetooth beacons, to track the spread of contagion in confined environments like healthcare facilities (23–26). However, these methods face challenges due to privacy issues, technological constraints, discomfort among patients and staff, and the inherent complexity of human movement patterns (27, 28). Relying solely on infectious disease testing to monitor contagion is problematic, as transmission can occur between test intervals, and test results are often subject to false positives and negatives. This necessitates robust metrics that account for the uncertainty in both location data and disease testing outcomes to improve the modeling and containment of infection spread within closed, interactive environments like healthcare settings.

**Figure 1:**
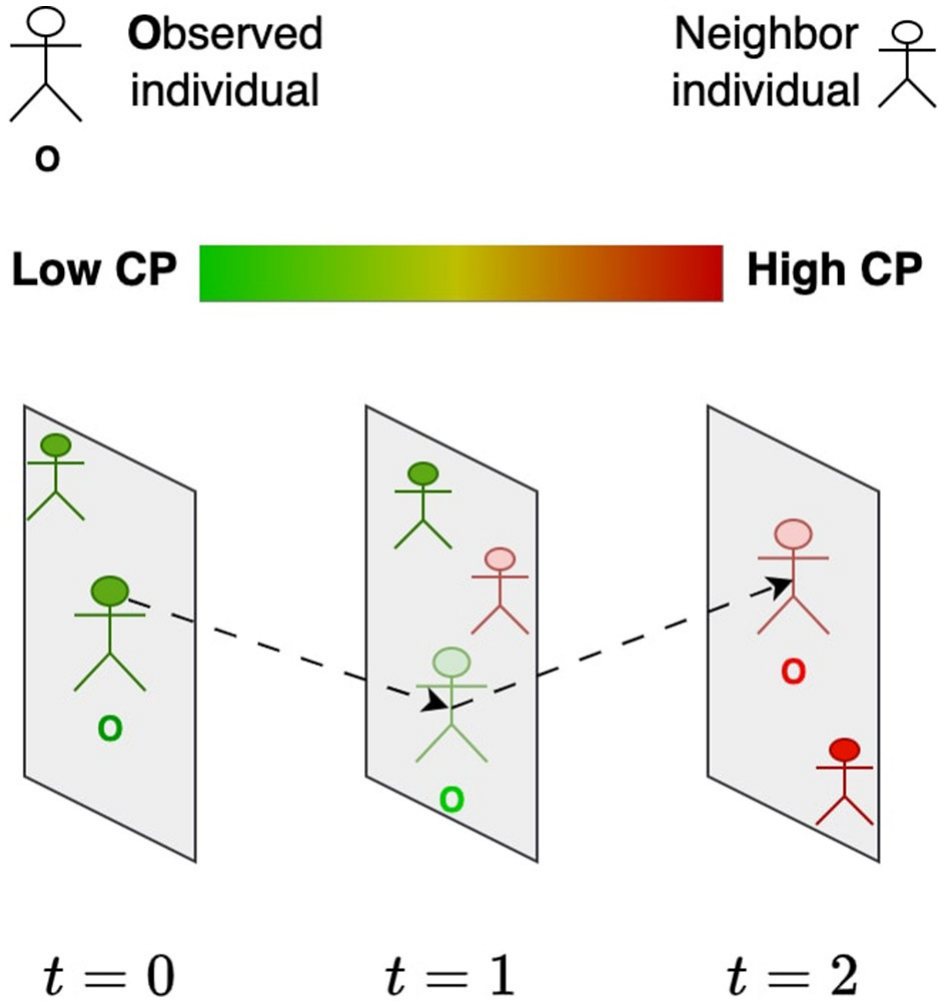
Evolution of the contagion potential (CP) of an individual (**O**) from low (green) to high (red) based on the CP of other individuals he comes in contact with over time.

We introduced the *contagion potential* (CP) as a continuous metric to quantify the overall infection risk contributed by both symptomatic and asymptomatic individuals (29, 30). *The concept of CP is grounded in the dynamic nature of infection propagation through social contact, where an individual's risk is influenced not only by their infection status but also by the CP values of their recent contacts*. Unlike traditional compartmental epidemic models that rely primarily on diagnosed infection states, CP provides a more granular, network-driven representation of transmission risk. Specifically, CP does not solely depend on whether an individual has tested positive or negative; rather, it evolves as a function of their exposure history and the infection risks of their immediate social network. As illustrated in Figure 1, an individual's CP transitions over time (*t* = 1, 2, 3, …), progressing from lower values (green, near 0) to higher values (red, near 1) based on repeated interactions with high-risk contacts. Prior work (31, 32) has demonstrated that CP integrates principles from network diffusion and optimization-based models, leveraging spatial contact structures and epidemiological properties to provide a robust estimate of individual infection risk.

In this paper, we leverage the contagion potential (CP) framework to reduce the incidence of hospital-acquired infections (HAIs). We evaluate infection risk at the individual level by accounting for heterogeneous characteristics and movement patterns of healthcare workers (HCWs) and patients within a facility, offering a dynamic and adaptable measure of both susceptibility and transmissibility. Healthcare facilities are composed of distinct *units*—such as hallways, triage zones, patient rooms, and waiting areas—between which individuals transition over time. Our framework, details presented in Section 2, models these interactions using approximate location information, represented as transition probabilities between units, thereby avoiding the need for fine-grained location tracking while still capturing the temporal and spatial structure of contacts. We introduce a dynamic learning model that continuously updates CP parameters based on observed transitions and infection data, enabling more accurate and timely risk assessments at both individual and unit levels. This risk-aware modeling is embedded within an optimization framework that, to the best of our knowledge, is the first to jointly tackle inpatient assignment in a way that minimizes the spread of HAIs while satisfying logistical and clinical requirements. Our current framework specifically focuses on modeling and mitigating the contact-based transmission dynamics of infection, particularly those acquired through interpersonal interaction and movement across spatial units. Infections arising primarily due to endogenous flora or medical devices (e.g., surgical site infections or catheter-associated infections) are governed by distinct clinical and physiological mechanisms that are not directly influenced by mobility or interaction-based contagion patterns. As such, while these types of infections are clinically significant, they fall outside the scope of the CP framework presented in this study. By concentrating on mobility-driven and contact-mediated HAIs, the proposed approach addresses a key subset of preventable infections where risk is strongly shaped by behavioral and spatial factors, making it amenable to intervention through network-aware decision-making and dynamic risk estimation.

To validate the proposed framework, we conducted two sets of experiments (refer to *Results* under Section 3). The *first set* focuses on evaluating the individual modules within the framework. Specifically, we demonstrate the effectiveness of learning CP from the approximate location of individuals within the unit, the iterative refinement of CP parameters, and the optimization of the patient-to-unit assignment problem. Furthermore, we demonstrate that optimizing based on CP, rather than relying solely on binary infection status, yields a lower infection count, as it captures the nuanced contributions to contagion from asymptomatic patients as well as the heterogeneity in infection risk. The *second set* of experiments involves an integrated evaluation of the entire framework. This integrated experiment leverages a realistic gravity-based mobility model that accurately captures indoor movement patterns and epidemiological parameters, simulating bimodal infection peaks observed in a healthcare setting. These experiments establish the efficacy of the framework in reducing infection risk and optimizing operational decisions in healthcare.

## 2 Methods

### 2.1 SIRS epidemic model

We employ the Susceptible-Infected-Recovered-Susceptible (SIRS) model to describe the progression of infectious diseases, following the formulation by Brauer and Castillo-Chavez (33). The population of individuals *N* is divided into three categories: susceptible (*S*), infected (*I*), and recovered (*R*). Susceptible individuals become infected through interactions with infected individuals at a transmission rate β, while infected individuals recover at a rate γ. The transmission rate β is the product of the basic reproduction number *R*_0_ and the recovery rate γ (34). Recovered individuals, in turn, may lose immunity and transition back to the susceptible compartment with a rate δ. These dynamics are governed by a system of ordinary differential equations, as defined in Equations 1–3, offering a representation of disease spread.


(1) $$\begin{array}{l} Ṡ=-\frac{\beta SI}{N}+\delta R \end{array}$$

(2) $$\begin{array}{l} İ=\frac{\beta SI}{N}-\gamma I \end{array}$$

(3) $$\begin{array}{l} Ṙ=\gamma I-\delta R \end{array}$$

To extend the framework, we incorporate a spatial variant of the SIRS model. Here, individuals are assumed to move within a geographical domain, and interactions that facilitate transmission occur when individuals are within a radius *r* of one another.

### 2.2 Contagion potential based on spatial contacts

Contagion potential (CP) is a continuous variable that quantifies an individual's infectivity, capturing both direct infection status and indirect exposure risks from their recent contacts within the network. CP evolves dynamically, reflecting the accumulated risk from interactions in a social network. The CP of an individual *u* at time *t*+1, with neighbors *v*∈**N**_*t*_(*u*), is defined as:


(4) $$\begin{array}{l} \mu_{t+1}\left(u\right)=\alpha \mu_{t}\left(u\right)+\beta \underset{v\in {\text{N}}_{t}\left(u\right)}{\sum }\mu_{t}\left(v\right) \end{array}$$

In the above equation, μ_*t*+1_(*u*) represents the updated CP of individual *u*, while $\underset{v\in {\text{N}}_{t}\left(u\right)}{\sum }\mu_{t}\left(v\right)$ aggregates the CP values of *u*'s contacts at the previous time step. The parameter α captures the persistence of CP over time, while β modulates the influence of social interactions on the evolution of CP. The infection transmission rate β is derived as *R*_0_×γ. This formulation reflects a fundamental principle of infection propagation: an individual's likelihood of transmitting disease is influenced not only by their infection status but also by the CP of those they interact with. Exposure to highly infectious individuals raises one's CP due to cumulative exposure effects, similar to the force of infection in classical epidemic models (35). The update rule ensures that CP accounts for network-driven risk amplification, where repeated interactions with high-CP individuals increase transmission potential over time.

To maintain interpretability and comparability, CP values are constrained within the range [0, 1] using the boundary condition: μ = max(0, min(1, μ)) after each CP update. This restriction is both mathematically and epidemiologically motivated. A CP of 0 represents no transmission potential, typically assigned to individuals unexposed to the pathogen, while a CP of 1 denotes maximal transmissibility, corresponding to individuals with peak infectiousness. Many epidemiological risk scores, including transmission probabilities and infection indices, naturally reside in this range to reflect real-world constraints on disease spread. Constraining CP in this way also facilitates its interpretation as a probabilistic measure of infectivity and ensures consistency across different populations and scenarios.

### 2.3 Quantifying infectivity using contagion potential

Assuming that infection spreads through contact between susceptible and infected individuals, the probability of encountering an infected individual is proportional to the fraction of infected individuals in the population, represented as $\frac{I}{N}$. Thus, the number of new infections, denoted by ν, depends on the number of susceptible individuals *S* and the infected fraction $\frac{I}{N}$, and is given by:


(5) $$\begin{array}{l} \nu =\beta \times \frac{I}{N}\times S. \end{array}$$

This formulation is consistent with classical mean-field epidemic models (36), where the force of infection is proportional to the density of infected individuals in the population. However, such models typically assume perfect knowledge of infection status, which is rarely the case in real-world epidemics. As discussed in Section 2.2, contagion potential (CP) measures an individual's infectivity as a continuous real value in the range [0, 1], accounting for both symptomatic and asymptomatic individuals. The mean CP across the population, denoted by μ (and used interchangeably with CP), serves as an alternative representation of the infection burden, particularly in scenarios where direct observations of *I* are limited. By incorporating CP into the formulation, the number of newly infected individuals at any given time becomes: ν = β × μ × *S*.

### 2.4 Modeling occupancy and contact within a healthcare unit

The contagion potential (CP) of each grid evolves based on the movement of personnel and the inherent uncertainty in their contact information within units. The CP of a grid at any given time is calculated as the average CP of individuals within that grid. Considering the modeled movement of individuals between grids, the CP of grid *i* at time *t*+1 can be expressed as: $\mu_{i}^{t+1}=\frac{\underset{j}{\sum }\omega_{j\to i}^{t}\times \mu_{j}^{t}}{\underset{j}{\sum }\omega_{j\to i}^{t}}$, where $\omega_{j\to i}^{t}$ represents the number of people moving from grid *j* to grid *i* at time *t*, and $\mu_{j}^{t}$ is the CP of grid *j* at time *t*. In practical scenarios, the precise locations of individuals within a unit may often be unknown or undisclosed due to privacy concerns. Given *n*_*j*_ is the number of individuals in grid *j*, *r* is the radius of influence, and *A*_*j*_ is the area of grid *j*, the expected number of neighbors for a person in grid *j*, in terms of its population density, is:


(6) $$e_{j}=\max\left(\frac{(n_{j}-1)\times \pi \times r^{2}}{A_{j}},0\right)$$

This expected number of neighbors can be used to predict the updated CP of an individual. The estimated updated CP of an individual *u* in grid *j* at time *t*+1 is given by: ${\tilde{\mu }}_{t+1}\left(u\right)=\alpha \times {\tilde{\mu }}_{t}\left(u\right)\;+\;e_{j}\times \beta \times {\tilde{\mu }}_{j}^{t}$, where α and β are scaling factors representing the contribution of individual and grid CPs, respectively. As mentioned in Section 2.2, if this value exceeds 1, ${\tilde{\mu }}_{t+1}\left(u\right)=1$.

### 2.5 Equilibrium condition of unit occupancy and CP

The contagion potential (CP) of a grid is derived from an inter-unit transition probability matrix **T**, where *T*_*ij*_ represents the probability of transitioning from grid *i* to grid *j*. Using a PageRank centrality approach (37), the equilibrium distribution of individuals across grids is described by the eigenvector equation: **T**·**E** = **λ**·**E**, where **E** = (ϵ_*i*_) represents the equilibrium distribution of individuals across units, that is, the rank of grid *i* times *N*. λ = 1 ensures that the expected population is stable. Given **W** = (ω_*ij*_) as the grid transition matrix, where ω_*ij*_ is the likelihood of movement from the category of grid *i* to the category of grid *j* based on simulated hospital mobility between these categories, the transition probabilities are: $T_{ij}=\frac{\epsilon_{i}\cdot \omega_{ij}}{\underset{j}{\sum }\epsilon_{i}\cdot \omega_{ij}}.$ Similarly, the CP vector of grids, denoted as **μ**, satisfies the eigenvector equation under steady-state conditions **T**·**μ** = λ·**μ**, where λ = 1. Let **μ**_*t*_ = (μ_1_, …, μ_*G*_) represent the grid-wise mean CP vector at time *t*. The temporal evolution of the CP is given by:


(7) $$\begin{array}{l} \mu_{t+1}=\mu_{t}\cdot \text{T}. \end{array}$$

As the system stabilizes, the population distribution and grid CPs converge to their equilibrium values. In the experimental results presented (see *Results* under Section 3), Equation 7 is used to estimate the CP of a grid using the PageRank process to determine **T**.

### 2.6 Dynamic learning of contagion potential parameters

The iterative learning model is designed to progressively refine the CP parameters for individual *u* by updating them based on real-time feedback and data. By continuously adjusting the parameters through a sequence of learning iterations, the model adapts to the individual's specific contagion dynamics, improving prediction accuracy over time. This approach ensures that the CP estimation is closely aligned with the individual's observed behavior and characteristics. In the equations below, *d*_*u*_ represents the discrepancy between the true CP and the CP estimated based on current parameters (α, β) for a person *u*, while $\bar{d}$ denotes the population mean discrepancy.

Recall from Section 2.2, that if a person is infected, their actual CP is 1. The discrepancy observed for an infected individual is then the difference between 1 and their predicted CP. The weighting factor *w*, typically between 0 and 1, determines the contribution of *d*_*u*_ relative to $\bar{d}$. The learning rate for a person *u* after τ_*u*_ updates is denoted by *lr*_*u*_(τ_*u*_), and the discounting factor for learning rate updates is represented by ξ, where ξ∈[0, 1]. The following update rule is used for all *v*∈{α_*u*_, β_*u*_:*u* = 1, 2, …, *N*}. The parameter *v* is referenced for convenience.


(8) $$\begin{array}{l} \text{Update Rule for}\text{ v}:\;v\leftarrow v+lr_{u}\\ \;\times \left(1-w\cdot d_{u}-\left(1-w\right)\cdot \bar{d}\right) \end{array}$$

(9) $$\begin{array}{ll} \text{Learning Rate Update:} & \;lr_{u}\left(\tau_{u}+1\right)\leftarrow lr_{u}\left(\tau_{u}\right)\cdot \xi \end{array}$$

The CP parameters are determined by sequentially running the α_*u*_ and learning rate updates, as follows. *First*, the update rule adjusts α_*u*_ based on the weighted sum of *d*_*u*_ and $\bar{d}$. The weight *w* determines the balance between individual discrepancy *d*_*u*_ and the mean or reference discrepancy $\bar{d}$. *Second*, the learning rate update decreases the learning rate *lr*_*u*_ over time to stabilize learning and prevent overshooting.

### 2.7 Patient assignment

The formulations below aim to minimize the overall risk associated with assigning individuals to different units, considering their specific requirements for specialized treatments.

#### 2.7.1 Optimization approach

The decision variables include *X*_*uj*_, a binary variable indicating if a person *u* is assigned to grid *j*. The inputs consist of (1) *R*_*uk*_, a binary matrix indicating if person *u* needs specialized treatment *k*; (2) *C*_*jk*_, another binary matrix indicating if grid *j* belongs to category *k*; and (3) *Z*(α_*u*_, β_*u*_, *X*_*u*_), a CP (or risk) function for person *u* based on α_*u*_ and β_*u*_. To ensure treatment needs are met, a person should be assigned to a grid where a treatment need can be met, though note that a person may need a variety of treatments, and should eventually be assigned to a grid where they can receive each necessary treatment. In practice, there may be only one grid *j* in which treatment *k* can be given, or multiple grids may support treatment *k*. Thus, this framework ensures inpatients receive the necessary treatment while minimizing contagion.


(10) $$Objective:\;\min\sum_{u=1}^{n}Z(\alpha_{u},\beta_{u},X_{u})$$

(11) $$\begin{array}{l} Subject\;to:\\ \;\sum_{j=1}^{m}X_{uj}=\sum_{k=1}^{K}R_{uk},\;\forall u\in \{1,2,\ldots ,n\} \end{array}$$

(12) $$\;\sum_{u=1}^{n}X_{uj}\leq c,\;\forall j\in T$$

(13) $$\begin{array}{l} \;X_{uj}\cdot C_{jk}\geq R_{uk},\;\forall u\in \{1,\ldots ,n\},\;\forall k\in \{1,\ldots ,K\},\\ \;\forall j\in \{1,\ldots ,m\} \end{array}$$

(14) $$\;X_{uj}\in \{0,1\},\;\forall u\in \{1,2,\ldots ,n\},\forall j\in \{1,2,\ldots ,m\}$$

The risk score, based on CP, is $Z\left(\alpha_{u},\beta_{u},X_{u}\right)=\overset{28}{\underset{j=1}{\sum }}\alpha_{u}\mu_{t}\left(u\right)+X_{uj}\beta_{u}\mu_{j}^{t}NE_{j}$, where α_*u*_μ_*t*_(*u*) measures the decay in the CP of individual *i*. If person *u* needs to go to grid *j*, *X*_*uj*_ = 1, and the risk associated with grid *j* is $\beta_{u}\mu_{j}^{t}\times N\times E_{j}$, since *N*×*E*_*j*_ is the predicted number of people in grid *j* which has average CP $\mu_{j}^{t}$. The objective, as defined in Expression 10, is to minimize the risk by assigning individuals to grids in a manner that optimally satisfies their health requirements. Constraint 11 ensures that each person is assigned to units that fulfill their treatment needs. Constraint 12 represents optional consideration to restrict the capacity of each treatment (non-waiting room) grid to *c*, where *T* is the set of treatment grids. Constraint 13 ensures that a person can only be assigned to grids equipped to provide necessary treatments. Lastly, Constraint 14 enforces binary assignment decisions of a patient to a grid.

#### 2.7.2 Greedy baseline

The mean CP and number of infections recorded under the optimization framework are compared to a greedy assignment process, which uniformly assigns patients to grids based on their treatment needs.

## 3 Results

We validate the framework through two sets of experiments. The first set demonstrates the functionality and performance of each core module in isolation (see Section 3.1.10), while the second presents an end-to-end evaluation that integrates realistic epidemiological and mobility parameters (Section 3.2). COVID-19 is used as a representative case study due to its continued relevance, well-documented contact-driven transmission characteristics, and the availability of structured data reflecting healthcare-associated risks. However, being a data-driven optimization approach, the proposed framework, given relevant data, is designed to be generalizable to other mobility-driven and contact-mediated HAIs beyond COVID-19. Table 1 summarizes the default parameter values used in the simulation and their application to the synthetic dataset. The term “true” refers to ground truth values for model components such as the number of neighbors, contagion potential (CP), and epidemiological variables. The discrepancy between these true and inferred values serves as a measure of predictive performance throughout our experiments.

**Table 1:** Default values of the epidemiological parameters.

**Parameter**	**Value**
Population size (*N*)	330 (48, 49)
Number of grids	28
Temporal resolution	Daily
Reproduction number, Delta, Omicron (*R*_0_)	3.2, 9 (50, 51)
Contact radius (*r*)	6 feet (52)
Probability *I* to *R* (γ)	0.05 (30)
Probability *R* to *S* (δ)	0.025 (30)
Probability *S* to *I* (β)	0.16 = *R*_0_·γ (30)
Viral load shedding rate (α)	$\frac{1}{8}$ (53, 54)
Initial proportion of susceptible individuals (*S*_0_)	0.8
Initial proportion of infected individuals (*I*_0_)	0.2

### 3.1 Study design

#### 3.1.1 Specialized units and inter-zonal movement

**Figure 2:**
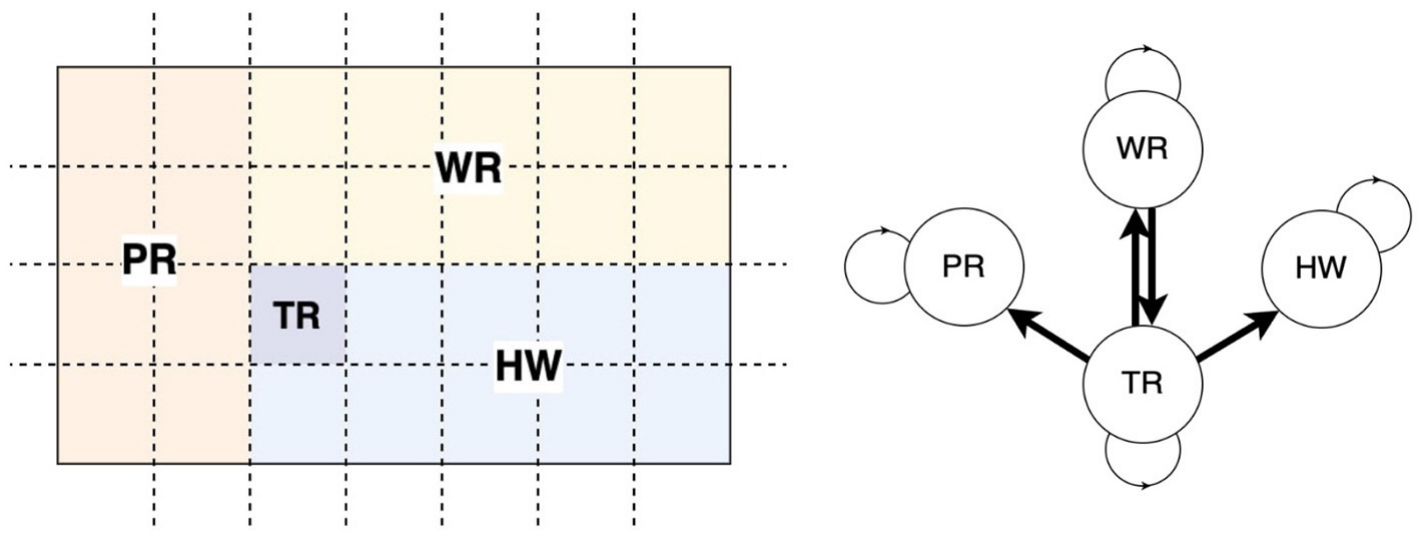
Healthcare units and movement of personnel. **(Left)** A facility is partitioned into four units, namely, patient room (PR), waiting room (WR), triage (TR), and hallway (HW). Each unit comprises grids (dotted squares). **(Right)** The clinical staff can move from one unit to another as per the directed links in the transition network.

We conceptualize specialized units within a healthcare facility as distinct *zones*, which are integral to infection control and patient management. As depicted in Figure 2 Left, these units include the *patient room* (PR), where individual care is delivered; the *waiting room* (WR), designated for patients awaiting treatment; the *triage* (TR) area, where the patient assessments occur; and the *hallway* (HW). Each section within a unit is modeled as a 10ft × 10ft grid square (38). The dynamic nature of patient and healthcare worker movement across these units contributes to varying levels of infection risk. A transition matrix (Figure 2 Right) is used to define the likelihood of individuals traveling between units. These probabilities provide a quantitative framework for evaluating infection risks due to movements.

#### 3.1.2 Three-phase contagion potential framework

**Figure 3:**
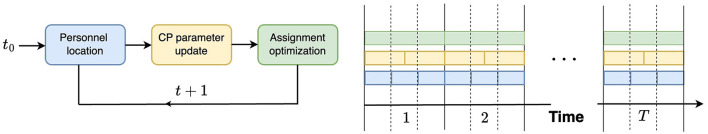
Study design: three modules of the contagion potential (CP) framework varying in frequency of occurrences: 1 (blue), high *H* (yellow), and low *L* (green).

The study is structured into three phases to address hospital-acquired infections (HAIs). First, we infer CP from approximate location data, leveraging the knowledge of the grid occupied by persons at a given time to estimate their infection risks. Second, we consider heterogeneous infection parameters α_*i*_ and β_*i*_ for individual *i*. As shown in Figure 3, we define three frequency levels (in decreasing order), 1, *H*, and *L*, such that 1 < *H*<*L*. Mobility occurs at the base frequency 1, CP parameter estimation is conducted at the intermediate frequency *H*, and patient assignment takes place at the lowest frequency *L*. Third, the optimization focuses on the grid assignment of patients and healthcare workers, using CP-based metrics to design optimal placement strategies that minimize infection spread while accounting for operational constraints. Optimized assignments are based on the CP of each grid. The CP of a grid is the average of the CPs of the individuals. Thus, the infection risk in a grid is higher when the CP of the grid is higher.

#### 3.1.3 Unimodal and bimodal infection trends

Classic disease models often consider one wave of infection, but as new variants arise in a pandemic or new countermeasures are implemented, multiple waves of infection occur. The bimodal incidence pattern has been specifically chosen because it more closely aligns with the contagion dynamics observed among SARS-CoV-2 positive staff and healthcare workers (39). When two waves of infection are considered, each wave corresponds to introducing a new variant. In the first (or only) wave, a variant with *R*_0_ = 3.2 is used, and 20% of the population is infected. This corresponds to the COVID-19 Delta variant. When a new variant is introduced, with *R*_0_ = 9, corresponding to the COVID-19 Omicron variant, 8%−25% of the population is infected. We perform experiments with both incidence models: Section 3.1.10, where we show the working of each module in the framework, is based on an unimodal incidence, while the complete analysis (in Section 3.2) uses a bimodal incidence.

#### 3.1.4 Generating an inter-unit transition matrix

We model the movement of individuals within the healthcare center with a classical gravity mobility model (40) that captures the complexities of movement better than homogeneous mixing. In the gravity model, the probability of moving from grid *i* to grid *j* is given by $\kappa \times \frac{n_{i}n_{j}}{d_{ij}^{\nu }}$, where *n*_*i*_ and *n*_*j*_ are the number of individuals in the grids *i* and *j*, respectively, *d*_*ij*_ is the distance between grids *i* and *j*, the constants κ = 0.08, ν = 1.78 as per (40). The inter-unit transition matrix is recalculated using the gravity mobility model since the number of individuals in a given grid evolves. The estimated values are row-normalized to ensure that each row in the transition matrix sums to 1. Thus, the resulting matrix is valid for stochastic modeling, effectively capturing inter-unit movement among the clinical staff.

We separately study the three aspects of the proposed CP framework: (1) the expected occupancy and contact among individuals within a unit (see *Methods* Section 2.4 and 2.5 for details); (2) updating CP parameters (see Section 2.6), and (3) the assignment of patients to units to control HAI (see Section 2.7.1).

#### 3.1.5 Expected unit occupancy

**Figure 4:**
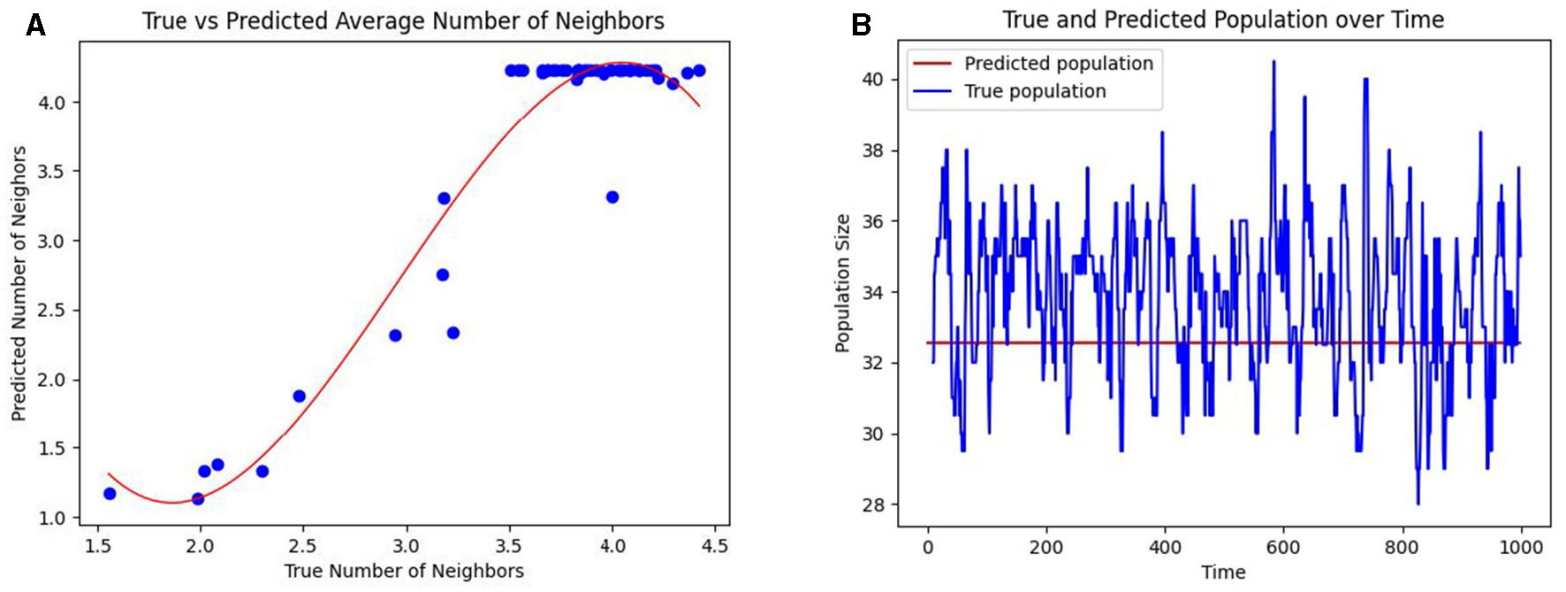
Prediction of the number of contacts per individual and the occupancy in healthcare units. **(A)** The mean predicted number of neighbors per individual (shown as blue dots) in a grid against the true mean. **(B)** The predicted occupancy (colored blue) and true occupancy (colored red) of a unit over time.

The *neighbors* of a person are the individuals they come into contact with. In a crowded unit with high population density, each occupant will likely have more neighbors and a commensurately high incidence of hospital-acquired infections (HAIs). Under the assumption of random mobility across and within units, the probability of moving from grid *i* to grid *j* is given by *t*_*ij*_, where *T* = (*t*_*ij*_). In this scenario, 330 individuals move about the healthcare facility. Under homogeneous mixing, we measure the number of contacts or neighbors of an individual as the expected number of people in a circle with a contact radius *r* = 6 feet. Over 100 hours, we calculate the average number of neighbors in a grid and compare it to our estimation of the expected number of neighbors (as per *Methods* Section 2.4). Figure 4A shows a strong cubic correlation (residuals 2.75) between the true and predicted neighbor counts. The fit line (shown in red) follows a polynomial of the form *y* = −0.61*x*^3^+5.41*x*^2^−13.81*x*+12.

Using a PageRank algorithm formulation of the transition matrix *T*, as per *Methods* Section 2.5, the PageRank of a grid, denoted as *E*_*i*_, measures the proportion of the total population in a given grid *i*. The expected population of grid *i* is then computed as *E*_*i*_×*N*, where *N* is the total number of individuals. This predicted population is compared to the observed population of a grid in Figure 4B. Together, these results suggest that even when precise mobility data is unavailable, the CP framework can infer interaction patterns based on the overall knowledge of the inter-unit transitions.

#### 3.1.6 Inferring CP from exact and inexact movement data

**Figure 5:**
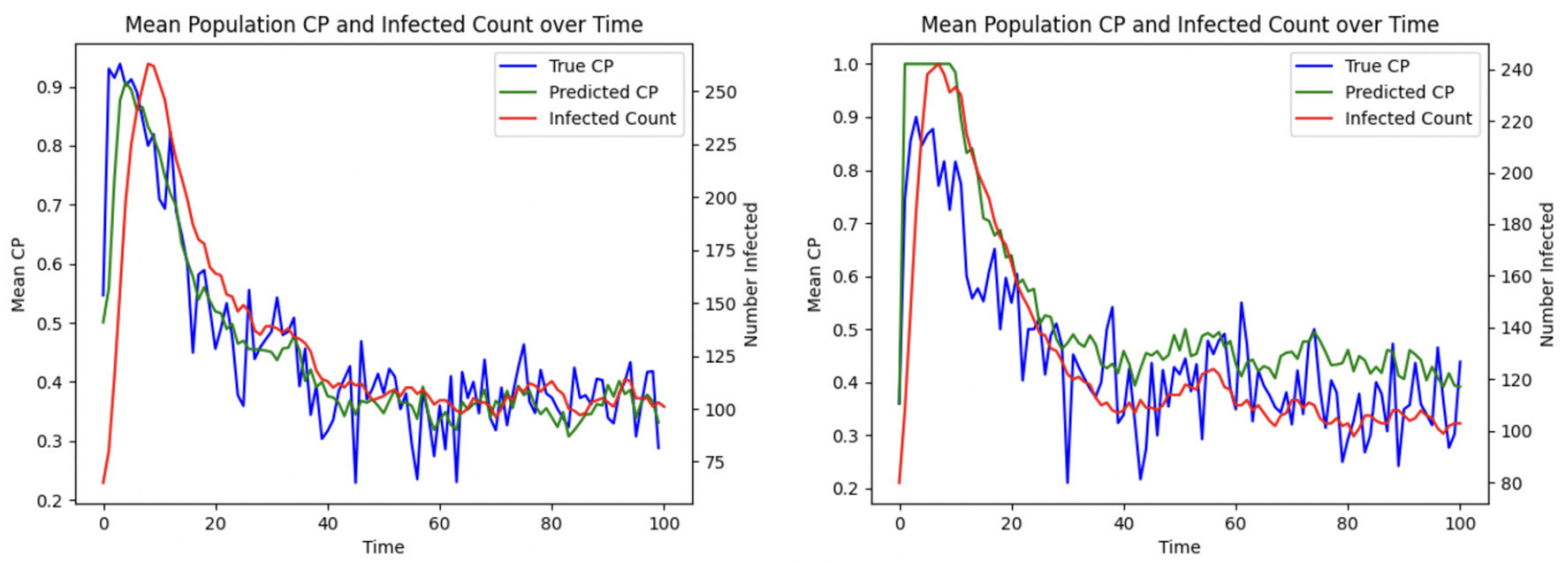
Inferring mean CP across healthcare units over time from exact and inexact movement data: **(Left)** Exact; **(Right)** Inexact.

Similar to the idea of estimating the occupancy of a healthcare unit, one can derive realistic estimates of CP under both exact and inexact knowledge of the number of people as per *Methods* Section 2.5. When the exact number of people entering grid *i* is unknown, *T* is used to predict the CP of grid *i*, as demonstrated in Figure 5 Left. Even with the added uncertainty surrounding the movement of individuals between units, we can closely predict the CP of a grid over time. Mobility is predicted based on the PageRank transition matrix, and Figure 5 Right shows that the predicted CP still closely follows the true CP of a grid.

#### 3.1.7 Inferring CP parameters

**Figure 6:**
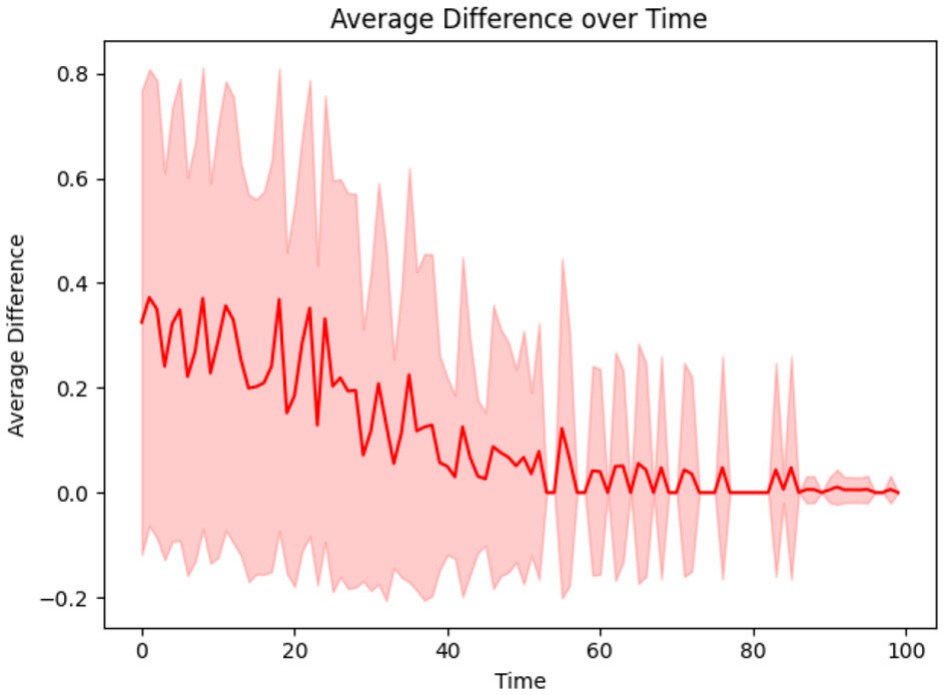
The difference between true and estimated infectivity of a person diminishes as their infectivity parameters are learned.

This aspect of the proposed CP framework focuses on minimizing the risk of infection for individuals by dynamically adapting their contagion parameters. Two key parameters are considered: α, which represents the rate at which an individual sheds the disease (with lower α indicating quicker shedding), and β, which quantifies the susceptibility of an individual to infection (with higher β indicating greater likelihood of acquiring the disease). Since these parameters are typically unknown for individuals, each *u* is initially assigned α_*u*_ and β_*u*_ values sampled from a distribution for all *u* = 1, 2, …, *N*. Over time, these parameters are iteratively refined using the update rules (described in *Methods* Section 2.6), which adjust α_*u*_ and β_*u*_ based on the discrepancies between the true and predicted infectivity levels of individual *i*. Equation 8 describes the update to α_*u*_ and β_*u*_, where the weighting factor between the current player's discrepancy and the average discrepancy is *w* = 0.2. The learning rate is then adjusted in Equation 9 with adjustment parameter ξ = 0.1. As the learning progresses, α_*u*_ and β_*u*_ converge to values that more accurately represent the true infectivity parameters of each individual, thereby minimizing the difference between the predicted and actual infection levels. This adaptive process is illustrated in Figure 6, which shows the reduction in the average discrepancy between true and estimated infectivity parameters across the population.

#### 3.1.8 Efficacy of CP in optimizing patient allocation

**Figure 7:**
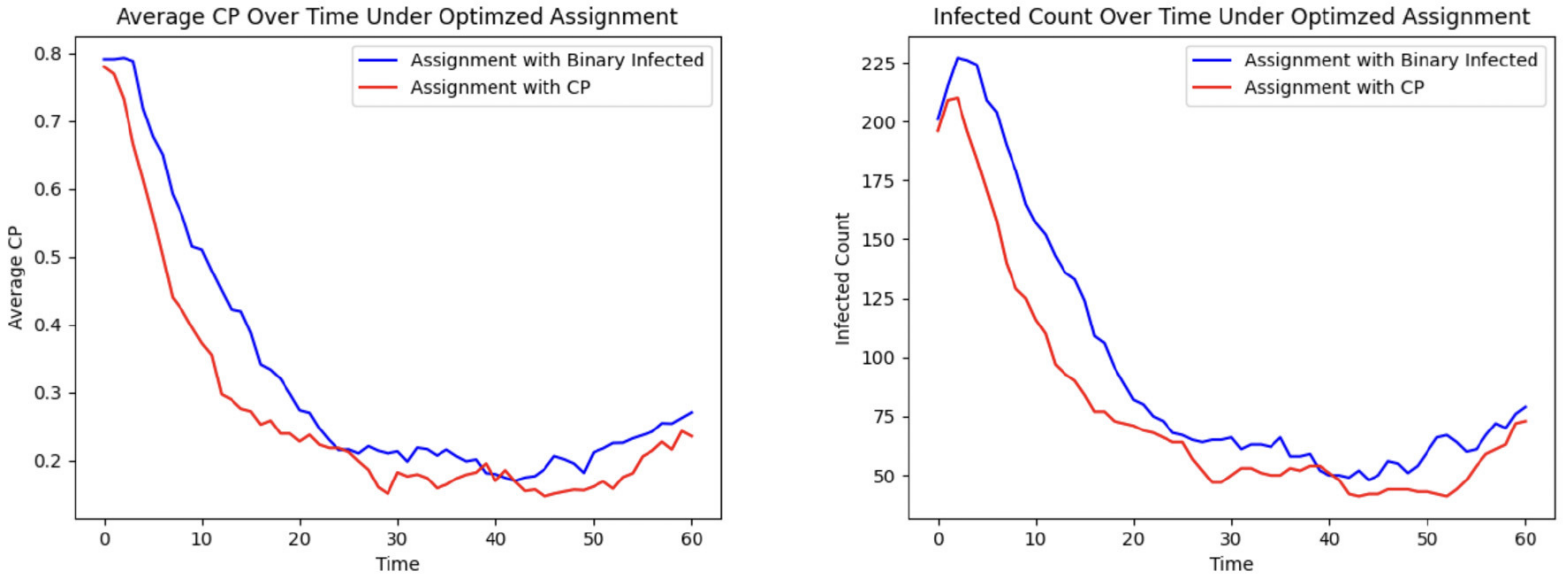
Patient allocation based on CP and SIRS model: **(Left)** mean CP; **(Right)** total infected.

To demonstrate the efficacy of CP in patient allocation, we conduct an experiment where the optimization formulation based on the SIRS epidemic model serves as a control, in which untested individuals are assumed to be uninfected. In contrast, our proposed CP-based optimization framework accounts for transmission potential, particularly from asymptomatic individuals who remain undetected. Figure 7 shows that the CP-based allocation reduces the mean population CP and the number of infected individuals over time. This improvement arises from the ability of CP to capture the hidden transmission dynamics of untested asymptomatic individuals, thereby highlighting its effectiveness in mitigating HAIs.

#### 3.1.9 Allocation of patients to units

**Figure 8:**
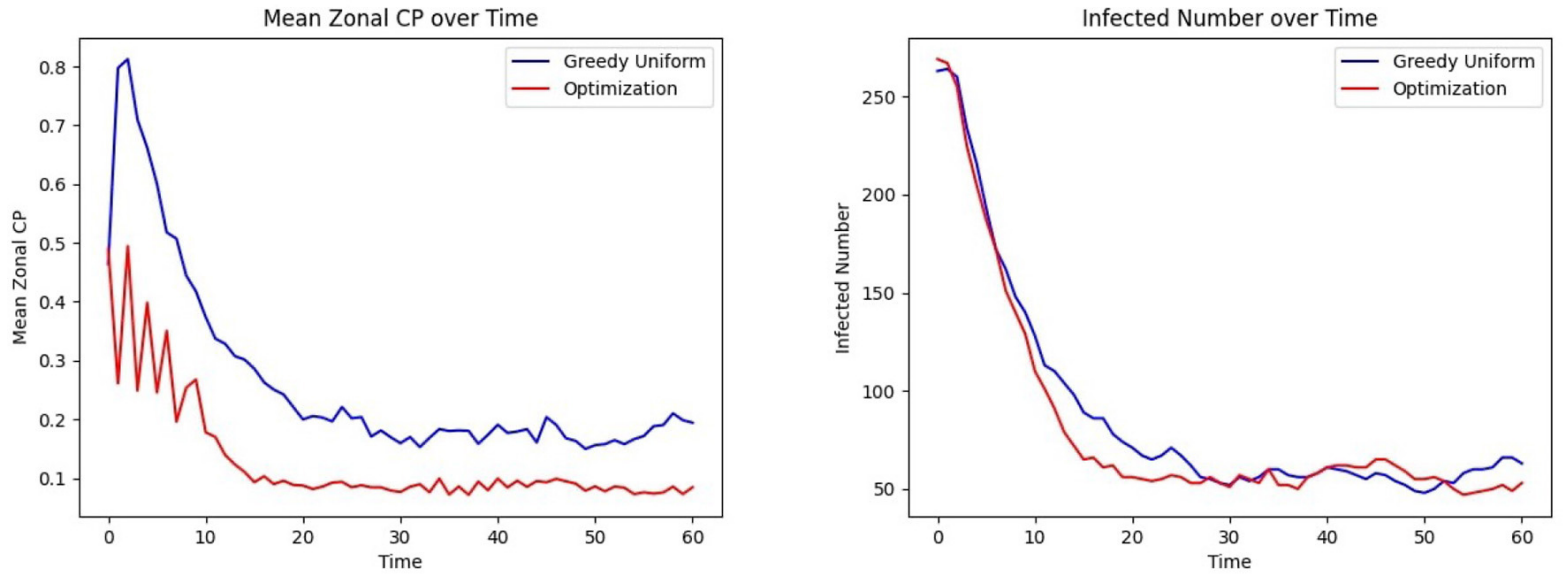
Effect of optimized assignment on infection spread under one infection wave: comparison of the **(Left)** mean CP **(Right)** infected number, under greedy and optimized assignments.

The last phase of the CP framework involves assigning patients to units, aiming to minimize their risk of exposure while ensuring their clinical needs are met. This is achieved through an optimization framework designed to mitigate infection trends (See *Methods* Section 2.7.1, where *R*_*uk*_ is a binary vector indicating that person *u* needs specialized treatment *k* and *C*_*jk*_ is a binary matrix indicating if grid *j* belongs to treatment category *k*). In this experiment, *C* is defined as a random 28 × 28 permutation matrix corresponding to the 28 grids, each with a treatment category, and each individual is assigned to visit between 2 and 14 grids. The CP-based risk of a person *u* moving to grid *j* at time *t* is calculated as α_*u*_×μ_*t*_(*u*)+β_*u*_×μ_*j*_, where α_*u*_ and β_*u*_ are the contagion parameters of individual *u*, μ_*t*_(*u*) represents their contagion potential, and μ_*j*_ denotes the average CP of grid *j*, estimating the exposure risk of grid *j*. The cumulative risk of the population is minimized while ensuring that individuals are assigned to grids capable of meeting their treatment requirements. The results of this optimization approach are compared to those obtained from a *greedy assignment baseline strategy* (discussed under Section 2.7.2). Figure 8 illustrates the outcomes of this procedure within an ER system.

The average grid CP is consistently lower for the optimization than for the greedy assignment, as shown in Figure 8 Left, indicating that the optimized assignment process effectively reduces the risk of infection. This is consistent with the result of Figure 8 Right. The number of infected individuals falls to equilibrium more quickly when assignments are selected to minimize CP than with the greedy assignment algorithm.

#### 3.1.10 Contagion potential for varying risk levels

**Figure 9:**
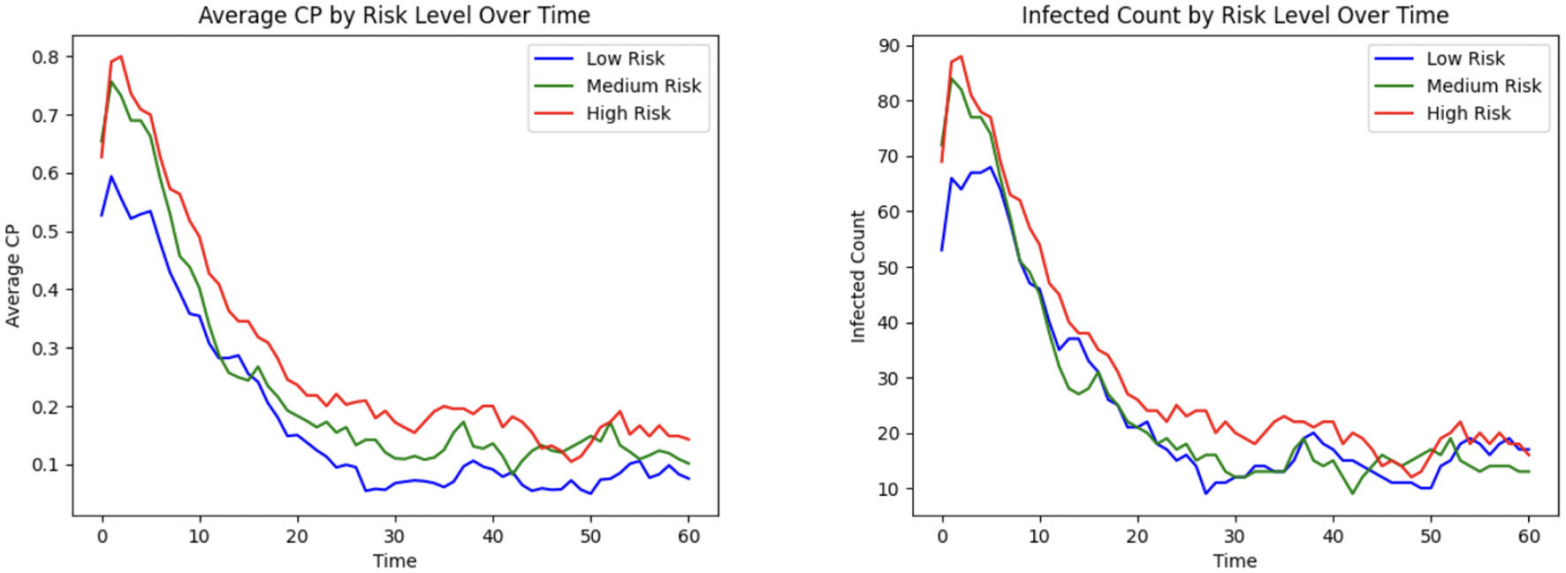
Infection risk and outcome based on high, medium, and low patient risk: **(Left)** mean CP; **(Right)** total infected.

CP provides a robust and adaptable framework for quantifying individual infection risk in heterogeneous populations. Figure 9 illustrates the dynamic evolution of average CP and infection counts over time across three risk strata, each consisting of 110 individuals: high-risk, medium-risk, and low-risk. These risk levels are encoded using distinct values of the susceptibility and transmissibility parameters (α_*u*_, β_*u*_), where high-risk individuals are assigned α_*u*_ = 0.8 and β_*u*_ = 2, medium-risk individuals α_*u*_ = 0.2 and β_*u*_ = 1.25, and low-risk individuals α_*u*_ = 0.05 and β_*u*_ = 0.5. As individuals move through different zones in the healthcare facility, the learning model (see Section 2.6) updates the CP parameters in response to observed transitions and infection data, allowing it to reflect the evolving infection dynamics adaptively. This stratified analysis shows that the CP metric effectively differentiates risk levels across population groups: high-risk and low-risk individuals consistently exhibit the highest and lowest average CP and infection rates over time, while the medium-risk individuals fall in between, in alignment with their parameterization.

#### 3.1.11 Effect of grid capacity

**Figure 10:**
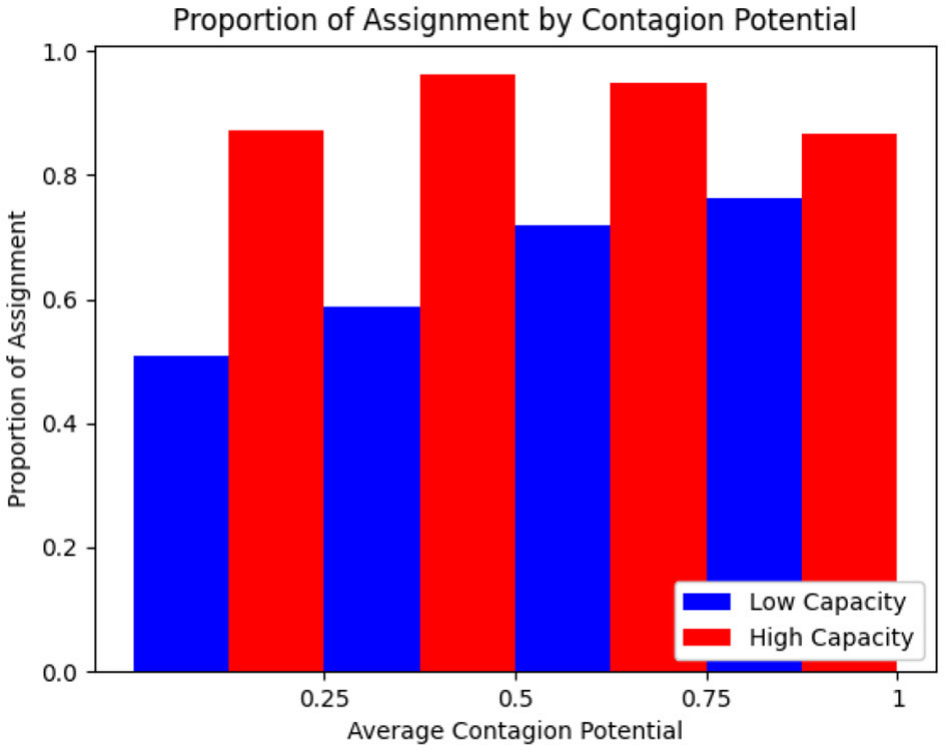
Proportion of rounds allocated to treatment based on mean CP for high and low capacity treatment rooms.

The optimization formulation for inpatient allocation to grids is designed to balance multiple objectives. Its effectiveness in minimizing HAI incidence is discussed earlier (see Section 3.1.9). We carry out an illustrative example to highlight how the allocation probabilities of patients vary in a small population of *N* = 50 based on CP under varying grid capacities, by considering a population of *N* = 50 and comparing two scenarios: high capacity (*c* = 30) and low capacity (*c* = 10). As depicted in Figure 10, where the CP values are binned and averaged over increments of 0.25, when capacity is high, most individuals, regardless of CP, are assigned to treatment grids with high probability. In contrast, under low capacity, individuals with high CP are prioritized for assignment, showing the ability to adaptively allocate limited resources by emphasizing contagion as a decision factor. This is consistent with the optimization seeking to isolate high-CP individuals so that their ability to infect others is limited.

### 3.2 Complete experiment

**Figure 11:**
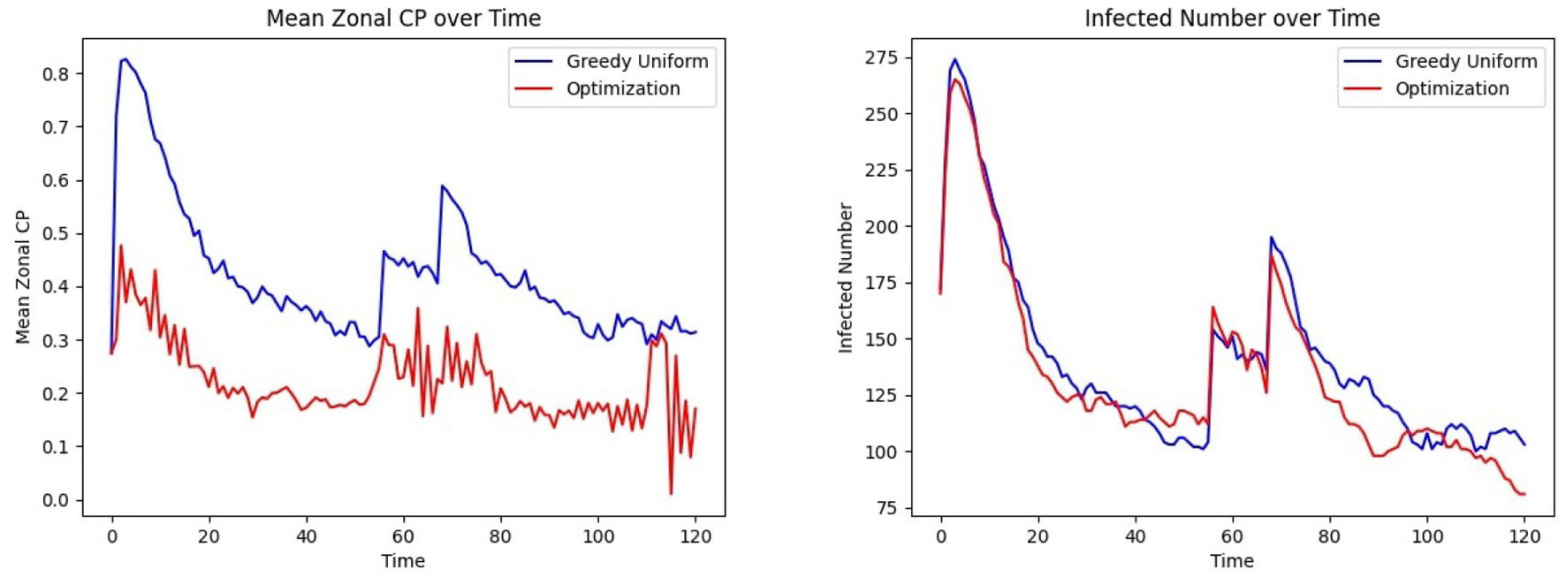
Effect of optimized assignment on infection spread under two infection waves: comparison of the **(Left)** mean CP **(Right)** infected number, under greedy, and optimized assignments.

This experiment integrates all the modules discussed in Section 3.1.10 into a comprehensive, end-to-end analysis of the proposed CP-based HAI control framework, incorporating realistic epidemiological and mobility trends. Specifically, we model the occurrence of multiple waves of infection within a healthcare setting, capturing the dynamic nature of disease spread. Figure 11 follows the same optimization procedure outlined earlier, now extended to account for a second wave of infection. This second wave is characterized by a strain with *R*_0_ = 9, compared to the initial wave's *R*_0_ = 3.2, reflecting the potential emergence of more transmissible variants. Furthermore, to achieve a realistic representation of patient and healthcare worker movement patterns, we employ the gravity model for indoor mobility (Refer to Section 3.1.4).

Figure 3 outlines the procedures involved in the complete experiment. At each time step, the location of individuals is updated, as described in the *Study Design* section (Section 3.1), which defines three frequency levels: 1, *H*, and *L* such that 1 < *H*<*L*. Recall that mobility occurs at the base frequency 1, CP parameter updates appear at the intermediate frequency *H*, and personnel assignments are optimized at the lowest frequency *L*. The CP updates are preceded by infectious disease testing, which provides ground truth for CP learning and updates, as *CP* = 1 is assigned to tested infected individuals. Although personnel allocation is desirable to control infection levels, these assignments occur less frequently than CP updates due to logistical constraints. This process is implemented over a simulated period of 120 h, with *H* = 3, *L* = 8, and location updates occurring three times an hour. Two waves of infection are studied during this period, consistent with the COVID-19 pandemic data analyzed in Lindsey et al. (39). Figure 11 Left, Right show that optimized patient assignment shows a reduced mean CP and daily incidence than the greedy patient allocation approach that relies on assignment based on treatment needs (Section 2.7.2).

**Figure 12:**
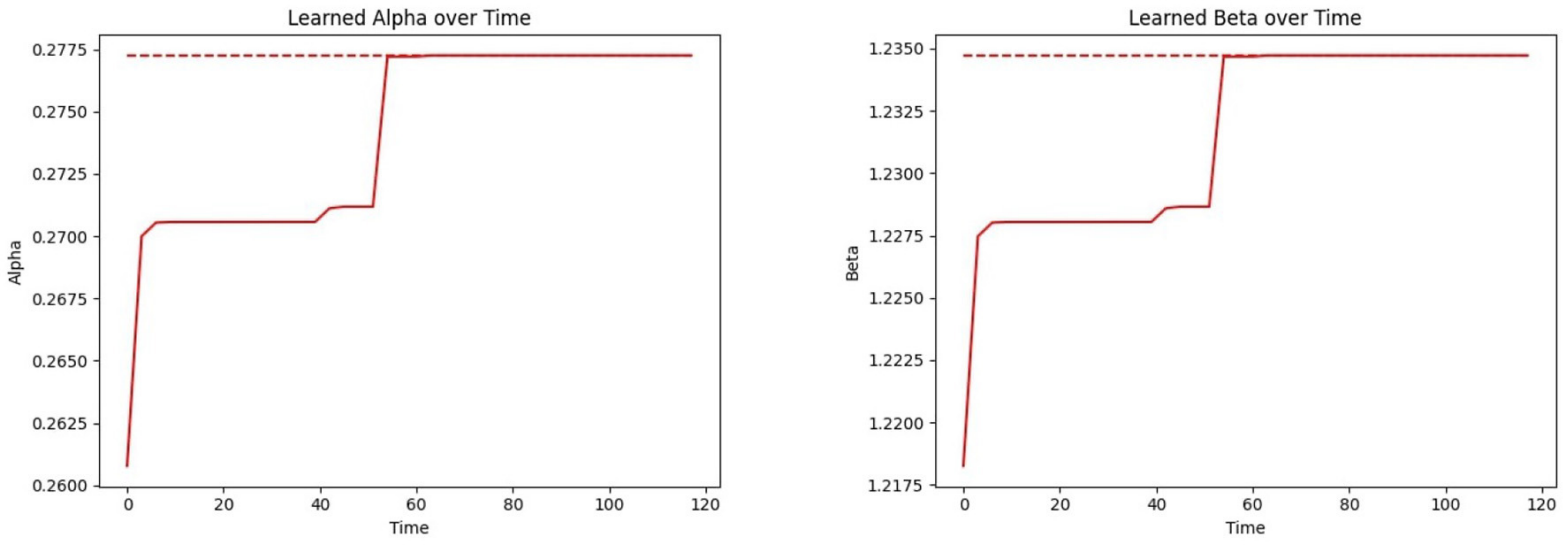
Convergence of the CP parameters α_*i*_ and β_*i*_ (inferring over time using the proposed learning model) to the true mean parameter values: **(Left)** CP parameter (α); **(Right)** CP parameter (β).

Figure 12 Left shows the convergence of the estimated mean α to the true mean α, and Figure 12 Right shows this process for β. Both parameters follow a similar trajectory as they are learned simultaneously through the same samples and procedures. Note that the time required for parameter convergence is highly dependent on the number of tested infected individuals. Since the learning model (see Section 2.6) relies on the infected individuals, a higher infection prevalence accelerates the learning process.

**Figure 13:**
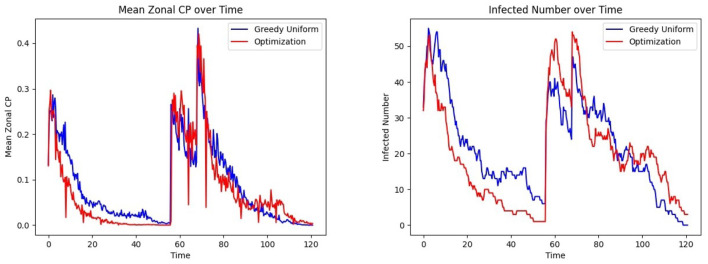
Effect of patient assignment on infection spread under the bimodal infection curve: comparing the **(Left)** mean CP and **(Right)** infected count under greedy and optimized assignments.

**Figure 14:**
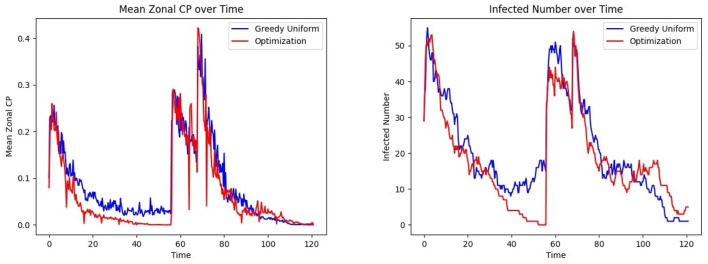
Effect of patient assignment on infection spread under the gravity mobility model: comparing the **(Left)** mean CP and **(Right)** infected count under greedy and optimized assignments.

Figure 13 Left shows that under the bimodal infection curves (refer to Section 3.1.3), the average CP remains consistently lower under the optimization protocol compared to the greedy algorithm, though the difference is smaller than when optimization is performed daily. Figure 13 Right depicts that infections decrease more rapidly under optimization, even when optimization is applied less frequently. Figure 14 gives the CP and infection trends for the gravity-model based mobility, showing that a similar circumstance of infection dying out more rapidly under optimized assignments.

## 4 Discussion

We proposed a contagion potential (CP) framework to address hospital-acquired infections (HAIs) by dynamically assessing infection risks based on individual characteristics and movement behaviors within healthcare facilities. CP employs macro-location data to provide a real-time infection risk landscape without necessitating precise tracking. This approach models interactions between patients and HCWs across key units, such as hallways, triage areas, and patient rooms, offering a practical and scalable solution for infection control. The framework further integrates a continuous learning model to refine CP parameters iteratively based on collected data, enhancing its capacity to assess risks accurately at both individual and unit levels. A key contribution of this work is the integration of CP within an optimization framework, making it one of the early efforts to allocate patients to healthcare units while simultaneously addressing objectives related to contagion mitigation, logistics, and clinical care. Unlike conventional patient allocation strategies that primarily focus on capacity constraints or clinical urgency, our approach jointly optimizes infection control while ensuring that logistical and clinical requirements are met. By embedding CP into the decision-making process, the optimization framework provides a dynamic and data-driven mechanism to guide patient assignments in a manner that reduces contagion risk while maintaining operational efficiency. This multi-faceted approach not only enhances the effectiveness of HAI mitigation strategies but also underscores the potential of computational frameworks in improving healthcare resource management.

Our analysis confirms the efficacy of the CP framework in approximating contact information to learn contagion dynamics. By avoiding the need for exact location data, the model leverages coarse-grained macro-locations to assess risks. An optimization framework for patient allocation based on CP is demonstrably more effective in minimizing contagion compared to the compartmental epidemic models, which assign a binary infection status to tested-positive individuals. The framework's adaptability, driven by its continuous learning capability, allows it to respond dynamically to shifting infection patterns and operational constraints, positioning it as a future tool for patient allocation while containing HAIs.

To model infections arising from endogenous flora and invasive medical procedures, the current CP framework could be expanded beyond its contact-based paradigm (41). This begins with interpreting CP as a composite risk score that integrates both interpersonal transmission risk and endogenous susceptibility. While the existing model evaluates infection risk based on movement patterns and interpersonal contact, endogenous infections depend more heavily on internal physiological conditions, prior colonization, immune status, and exposure to procedures such as catheter insertion or surgery (42). Incorporating these factors requires augmenting the CP calculation with patient-specific clinical features drawn from electronic health records, as well as procedural exposure histories. This integration could result in a hybrid contagion metric that more accurately reflects multimodal infection risk in healthcare settings stemming from myriad multidrug-resistant organisms. A second critical adaptation involves the temporal modeling of procedure-related risk. Many endogenous infections follow time-sensitive trajectories post-exposure—e.g., surgical site infections peak within days after surgery, and catheter-associated infections increase with dwell time (43). The framework must further include explicit timepoints for high-risk procedures and dynamically adjust the CP score over time as procedural windows open or close. Lastly, the existing optimization framework (as discussed in Section 2.7.1), which currently assigns patients to units by minimizing contagion while satisfying logistical constraints, must be reformulated as a joint optimization problem. This new formulation must simultaneously minimize risks from both contact-based transmission and endogenous sources, potentially involving trade-offs between spatial cohorting and procedural scheduling. By addressing these three adaptations, the CP framework can evolve into a tool for infection prevention across diverse etiologies and healthcare scenarios.

The framework also has implications for patient privacy and the secure use of clinical data. Specifically, the information shared within the CP-based optimization framework consists of patient location data and clinical needs for allocation to the appropriate grid. By focusing on macro-location tracking rather than precise geolocation, our approach lends itself to privacy-preserving features, such as group-based anonymization techniques like k-anonymity and l-diversity (44). Therefore, a detailed investigation of privacy-preserving safeguards is part of our ongoing research efforts. However, emerging privacy-preserving technologies, such as multiparty homomorphic encryption (45) and differential privacy (46), offer promising avenues for future exploration. For instance, differential privacy could be employed to aggregate CP at the grid level without exposing individual health indicators, while federated learning could enable collaborative data modeling across institutions without sharing raw patient data (47).

## Data Availability

The datasets presented in this study can be found in online repositories. The names of the repository/repositories and accession number(s) can be found below: https://github.com/satunr/HAI/.
